# Successful Management of Acute Catastrophic Juvenile Vaginal Bleeding in Glanzmann's Thromboasthenia by Uterine Tamponade: A Case Report and Review of The Literature

**DOI:** 10.1155/2012/530908

**Published:** 2012-03-20

**Authors:** Nazli Hossain, Tahir S. Shamsi, Adeel Feroz

**Affiliations:** ^1^Department of Obstetrics & Gynecology Unit 3, Dow University of Health Sciences, Baba-e-Urdu Road, Karachi 74200, Pakistan; ^2^National Institute of Blood Disease & Bone Marrow Transplantation, Karachi, Pakistan; ^3^Dow University of Health Sciences, Baba-e-Urdu Road, Karachi 74200, Pakistan

## Abstract

Glanzmann's thromboasthenia (GT) is a rare platelet disorder, due to membrane defects involving glycoprotein GP IIb/IIIa complex. Symptoms appear in infancy with episodes of bruising, gingival bleeding, epistaxis, or at the time of menarche acute episode of uterine bleeding. Hormonal therapy and antifibrinolytic agents are first-line treatment. Platelet transfusion is given to control hemorrhage when medical treatment fails. However, repeated transfusions may result in development of platelet refractioness, due to development of antibodies against membrane glycoprotein. Activated recombinant FVII is licensed for use in acute control of bleeding in GT. Here we report a case of acute juvenile uterine bleeding at menarche, which responded successfully to uterine tamponade. To our knowledge, this is the first case report on use of balloon tamponade for control of acute catastrophic juvenile bleeding at menarche.

## 1. Introduction

Glanzmann's thromboasthenia (GT) is an autosomal recessive disorder, with reported incidence of 1 in 100,000. Increased prevalence of the disease is seen in Jewish population and in communities with increased incidence of consanguineous marriages. The defect involves glycoprotein complex IIb/IIIa complex, resulting in failure of platelet aggregation [1]. There is a history of bruises, epistaxis, bleeding from gums, and profuse vaginal bleeding at menarche [2]. Diagnosis is usually made in infancy for these symptoms, or at the time of menarche. Transfusion of platelets is the standard form of treatment; however, refractoriness to platelet transfusion due to formation of antibodies is reported. Activated recombinant factor VII is licensed for use in GT. We have earlier reported on use of rFVIIa for platelet function disorder in adolescent menorrhagia [3].

Here we report a case of acute catastrophic vaginal bleeding in a girl, who was diagnosed with GT and responded to uterine tamponade for acute control of vaginal bleeding.

## 2. Case Report

A 12-year-old girl was seen in the emergency room, with complaints of heavy bleeding per vaginum, since last 15 days, along with dysmenorrhea. She was referred to us from basic health unit. According to her mother, this was her first menstrual period. She had 7 siblings, 4 sisters, and 3 brothers. Her two elder sisters had normal, regular periods. Her past history was significant for bruises, epistaxis, and bleeding from gums from the age of three years. She was never transfused blood for these complaints, but did receive medical attention for the above complaints of epistaxis and bleeding from gums. One of her cousins had also died at the age of eight years, from uncontrollable hemorrhage from mouth and nose. Her maternal grandfather also died of intracranial hemorrhage, but there was no definitive diagnosis in both of the above deaths.

At the onset of menarche, she suffered from heavy blood loss, with passage of blood clots. The amount of blood loss resulted in transfusion of one unit of blood. She was also kept on injectable tranexemic acid. The bleeding decreased initially for 48 hours, but was later followed by heavy bleeding per vaginum. At the onset of second episode of heavy vaginal bleeding, she was referred to Civil Hospital Karachi, for further management.

General examination revealed extreme pallor, with blood pressure of 90/60 mm hg. Physical examination was unremarkable. Local examination revealed soiling of perineum, with blood and blood clots. Her hemoglobin concentration was 6 gm/dL, platelets count was 200,000, and prothrombin and partial thromboplastin time was normal. Her pelvic ultrasound was normal, with evidence of hematocolpos. She was started on injectable tranexemic acid 500 mg eight hourly, along with tab norethisterone 5 mg, three times daily. A single injection of depot medroxy progesterone 150 mg was also given. She was transfused four units of packed red blood cells. Her bleeding continued, despite the above measures. Her factor VIII level was assessed to rule out vWD and was found to be 150%. On the seventh day of her admission, her hemoglobin dropped to 3.8 gm/dL, with platelet count of 178,000. At this point we decided to perform platelet aggregation studies. Reports showed the following: ADP 18.1 (76.36–106.84%), collagen 20.4 (59–102%), epinephrine 24.1 (73.7–109.3%), ristocetin 59 (70.4–111.2%), and arachidonic acid 12.9 (55–127%). A diagnosis of Glanzmann's thromboasthenia was made. She was transfused with one mega unit (single-donor apheresis) of platelets then bleeding slowed for 6 hours and started again. She further received 4 more doses of platelets, but there was no improvement in bleeding. At this point, a decision was made for rFVIIa. She was given rFVIIa in a dose of 120 *μ*g/kg, dose repeated after 4 hours; bleeding slowed down a bit but continued. Subsequent two doses of 270 *μ*g/kg were given during next 24 hours. Bleeding decreased for 48 hours, but continued, and her hemoglobin again dropped. A decision for uterine tamponade was taken. Examination under anesthesia was done, and two Foleys catheters of size 10 F were passed in the uterine cavity and were inflated with 30 cc of normal saline to act as tamponade. They were left inside for 48 hours; then, bleeding stopped completely. She stayed in hospital for next few days and was discharged on oral contraceptive pills and ferrous sulphate.

## 3. Discussion

Abnormal uterine bleeding in adolescent is commonly seen due to immature hypothalamic pituitary axis, polycystic ovarian syndrome, or thyroid disorders. Exclusion of these common disorders should be followed by exclusion of inherited bleeding disorders. The prevalence of inherited bleeding disorders in adolescent menorrhagia has been reported between 4 and 48% [4–6]. These include idiopathic thrombocytopenic purpura, vonWillebrand's disease, and platelet aggregation defects. Few cases of adolescent menorrhagia have been reported because of platelet aggregation defects, including Glanzmann's thromboasthenia [7–9]. This platelet disorder has been commonly reported amongst French gypsies, Iraqi Jews, Arabs of Jordanian decent, and Indians [10, 11]. This has been attributed to consanguineous marriages. Increased frequency of bleeding disorders due to consanguineous marriages has earlier been reported from our region as well [12].

The diagnosis of GT is usually made in infancy, or at the time of menarche. Management options for menorrhagia include supportive treatment in form of transfusion of blood and blood products, hormonal therapy, balloon tamponade, and activated recombinant factor VII, which is licensed for control of massive hemorrhage. Our patient also received massive transfusion of blood and blood products (10 units of packed red cells, 5 doses of platelets transfusion). She also had a history of transfusion of blood and blood products in past. This may have been the reason for her refractoriness for platelet transfusion. If medical treatment fails, transfusion of platelets is considered as the first-line treatment, once the diagnosis of GT is made. Continuous heavy bleeding per vaginum resulted in fall in hemoglobin to 3.5 gm/dL, and required administration of rFVIIa in higher doses to stop bleeding. Hormonal therapy includes high doses of intravenous estrogens for acute control of blood loss, followed by maintenance therapy with combined oestrogen and progesterone preparations. We do not have injectable preparations of oestrogen available; hence a depot form of progesterone was given. This was our first experience with use of balloon tamponade for acute control of juvenile bleeding. Although this procedure has been extensively reported for postpartum hemorrhage, its use has not been frequently described for acute control of juvenile bleeding. It is recommended to pass Foley's catheter, of smaller size, and inflate it with at least 30 cc of saline to exert pressure. It is also recommended to do an ultrasound to see if there is a collection of blood above the tip of catheter [13]. The catheter is left inside for 24 hours. We left the catheter inside for 48 hours, and antibiotic cover was provided. Uterine artery embolization has also been reported for acute control of life-threatening hemorrhage, due to inherited bleeding disorder [14]. This may be done as a last resort before proceeding to hysterectomy.

There are reports of successful allogenic hematopoietic bone marrow transplant for GT, in cases of early life-threatening events [15].

Hormonal therapy is the main stay treatment in young girls, not desiring conception. Combined oral contraceptive pills are recommended to avoid heavy monthly blood loss. They may be combined with oral tranexemic acid and blood products during withdrawal period. Other options include cyclical progesterone therapy from 5 to 25 days of menstrual cycle, danazol, and GnRh analogues. The last is recommended only in cases of life-threatening bleeding episodes.

## 4. Conclusion

Inherited bleeding disorders are an important cause of adolescent menorrhagia. Gynecologists and family physicians must be aware of this as an important cause of bleeding at menarche and should also be aware of treatment modalities available. Intrauterine balloon tamponade is an inexpensive, important tool in acute control of juvenile bleeding and should be used earlier in treatment algothrim in low-resource settings.
